# Estimation of Diameter of Quadrupled Hamstring Graft for ACL Reconstruction using Pre-operative MRI Measurement as a Predictive Tool

**DOI:** 10.5704/MOJ.2403.012

**Published:** 2024-03

**Authors:** RS Kuruvilla, C Gunasekaran, TS Jepegnanam, M Kandagaddala, J Panwar

**Affiliations:** 1 Department of Orthopaedics, Christian Medical College, Vellore, India; 2 Department of Radiology, Christian Medical College, Vellore, India; 3 Department of Radiology, Lumus Imaging, Brisbane, Australia

**Keywords:** ACL, diameter, hamstring, MRI, prediction

## Abstract

**Introduction:**

The diameter of the quadrupled Hamstring graft plays a significant role in the incidence of graft failures for ACL reconstruction. The ability to predict the graft size pre-operatively can prepare the surgeon for alternatives in the event of an inadequate graft diameter.

**Materials and methods:**

We retrospectively measured the diameter of the Semitendinosus tendon (ST) on the MRI in all patients who underwent arthroscopic ACL reconstruction using quadrupled Semitendinosus as their graft. We also estimated any correlation between various anthropometric data with pre-operative MRI based Cross Sectional Area (CSA) of the Hamstring tendon and final graft diameter in the South Asian population. The patients were included from Jan 2018 - Dec 2020.

**Results:**

The minimum CSA of ST to predict an eventual graft diameter of 7.5mm was 10.7mm2. The MRI based cross-sectional area measurement showed moderate correlation with the intra-operative graft diameter obtained. (r=0.62, p<0.001). The intra-class correlation coefficient between the radiologist and the surgeon was 0.82, 95% CI (0.57, 0.92) and a p-value <0.001.

**Conclusion:**

Pre-operative MRI can be a useful tool to predict the graft diameter. This coupled with the anthropometric data of the patient can be used as an adjunct to estimate the probable graft diameter. Thus, the surgeon can be better prepared for the surgery and can seek alternate graft options if the graft size is deemed inadequate pre-operatively.

## Introduction

Anterior Cruciate Ligament Reconstruction (ACLR) is the accepted procedure for an Anterior Cruciate Ligament (ACL) deficient knee^1^. The autogenous choice for graft is wide, ranging from Bone Patellar Tendon Bone, Hamstrings, Quadriceps tendon, Peroneus Longus tendon. The two widely accepted choice for autograft is the Hamstring tendon and BTB. The Hamstring graft offers less donor site morbidity as compared to the latter, such as post-operative anterior knee pain^2^. The method of harvesting the Hamstring graft and its preparation techniques may not yield a uniform tendon thickness every time. This unpredictability can lead to alternate graft choices or augmentation methods intra-operatively.

One major limiting factor of using the autogenous Hamstring graft remains the variability in the graft diameter. Grafts with a diameter less than 7mm have a higher propensity for failure and revisions, therefore surgeons must aim to keep 7mm as the reference point during ACLR^3-7^.

Schlumberger *et al* did not find any significant difference in the revision rates of grafts having diameters <8 or >8mm for four-strand Hamstring grafts used in ACLR^6^. A graft diameter that has been described to be inadequate was taken as 7mm^8,9^. If the potential graft diameter could be predicted before the patient is on the table, it could help the surgeon plan the surgery better or seek alternate graft options or graft preparation methods.

Numerous studies investigate various methods to predict graft size pre-operatively. As almost all patients undergoing ACLR would have a pre-operative MRI for the diagnosis, this valuable tool could also be used to predict the eventual graft diameter. Ultrasound was proved to be an unreliable tool to predict and adds to the cost. MRI was used in studies and has shown to be a better predictor too^10-12^. A review of the literature found that in similar studies, the method of quadrupled hamstring tendon uses both the Semitendinosus (ST) and Gracilis tendon (GT). Quadrupled Hamstring (QH) tendon was studied by Vardibasis and found that MRI can accurately predict the final QH tendon diameter^9^. Our method of ACLR similarly uses only ST tendon, which was harvested through a posterior knee crease incision and subsequently quadrupled as a continuous loop and prepared by the Graft-Link method described by Lubowitz^10^. Thus, this technique offers more precision if it is reliably able to predict the eventual graft sizes as it measures only one tendon.

Multiple articles show the correlation of anthropometric data and the prediction of graft size. Direct correlation between anthropometric data and pre-operative MRI based prediction has been studied by Thwin *et al* and concluded that MRI is a better predictor than anthropometric data^13^. By combining the anthropometric data with the pre-operative estimation of graft size, the predictive value may be enhanced.

Hamada *et al* found a slight correlation between the patients’ height and CSA of ST measured on the pre-operative MRI with the length of Hamstring tendon harvested^11^. The patients’ height and gender can be used as pre-operative indicators of in vivo QH graft diameter concluded by Ma *et al*^12^.

Ethnic differences in the size of the tendon have been shown by a study published by Goyal *et al*. He concludes that the Indian demographic population will give a diameter often lesser than 8mm^14^. Our study was conducted on a similar demographic population.

The primary purpose of this study was to show whether pre-operative MRI could be used to predict accurate graft sizes using cross-section analysis (CSA) of the ST tendon. The second objective was to show a cut-off for the graft diameter and CSA below which the autogenous ST would not yield a graft diameter of 7mm or more. Furthermore, this study will test the inter-reader reliability of measuring the CSA of ST on the pre-operative MRI. The correlation of anthropometric data alone and in combination with the MRI estimation has also been studied to see whether the predictability of the diameter of the graft could be enhanced.

## Materials and Methods

**Fig 1:**
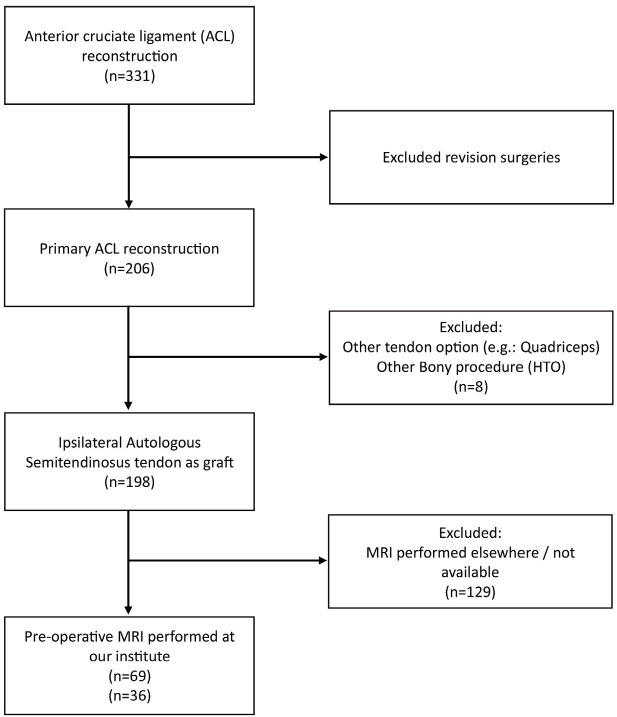
Patient search and workflow.

After obtaining prior IRB approval, retrospective data of all patients that underwent ACLR using Quadrupled ST from January 2018 – December 2021 were reviewed. Search for ACL / anterior cruciate surgeries yielded 267 patients. There were only 206 patients who underwent primary ACLR using autogenous quadrupled ST as their graft. After going through the records, only patients who had performed their pre-operative MRI here at our institution were included. The exclusion criteria included a prior operation on the knee, revision ACLR and other graft options such as quadriceps or BTB and ST harvested from the unimaged knee. This left us with 69 patients that were included in the study (Fig. 1). The demographic details and anthropometric data were collected from their hospital records. All the surgeries were performed by a single senior surgeon at our institute.

All patients in this study that underwent ACLR the graft of choice was autogenous ipsilateral quadrupled ST tendon. The graft was harvested through a posterior knee crease incision. The tendon was identified, and an open tendon stripper was used to extract the proximal portion of the tendon. Direct visualization and release of the vincula were done, and the distal portion was taken along with a sleeve of periosteum from its insertional site using a closed tendon stripper.

**Fig 2:**
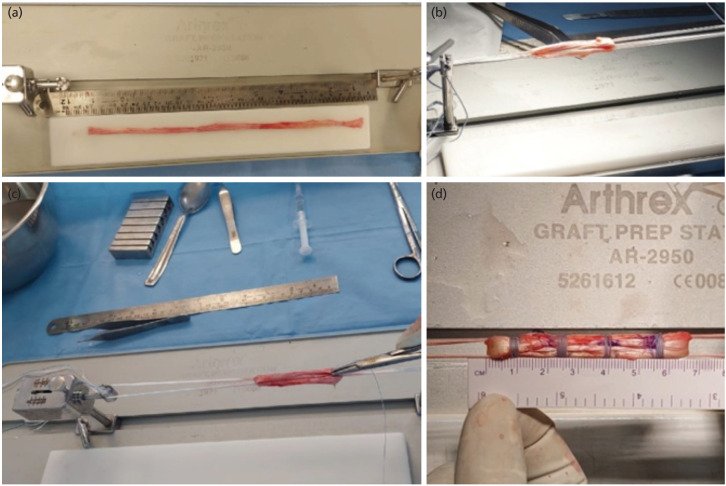
Graft preparation method (Graft-Link). (a) The full length of the graft. (b) The graft after looping 4x and an end stitch. (c) The linking stitches. (d) The Final quadrupled and linked graft.

Graft preparation was done by the Graft-Link technique^10^ (Fig. 2). The length measured and the tendon quadrupled as a continuous loop. It was ensured that the total graft length after quadrupling must be at least 5cm (this was done to ensure that at least 1.5cm of graft would be available in the femoral socket assuming that the intra-articular length would be around 2cm). Sizing of the graft was then performed using the calibrator with 0.5mm increments. ACLR was done through a standard single bundle all inside technique with suspensory fixation [Tightrope™ Arthrex, USA] on both the femoral and tibial ends. In cases where the graft size was deemed insufficient, as in high demand individuals with graft size less than 7mm INTERNAL BRACING was performed using a FIBERTAPE [Arthrex, USA] threaded through the femoral button and anchored on the tibial end using an independent fixation device [Swivel Lock™ Suture Anchor, Arthrex, USA]^15^. Meniscal tears in these individuals, whenever present, were addressed prior to the ACLR.

Standard post-operative protocol was followed and was tailored if ACLR was combined with meniscal repairs or PLC reconstruction. The MRI studies were performed with either a 1.5T [Siemens Avanto Fit] or 3.0T [Philips Achieva] using a dedicated knee-coil. The sequences acquired were Proton Density Weighted (PDW) and Proton Density Weighted-fat suppressed (PDW-FS) sequences in axial, coronal, and sagittal planes. The acquisition parameters were PDW-FS (TE/TR 32/3630; echo train length 7; matrix 240x320) and PDW (TE/TR 33/2950; echo train length 7; matrix 284x388). All the images were acquired with field of view 140x140mm, slice thickness 3.0mm and inter-slice gap 0mm).

**Fig 3:**
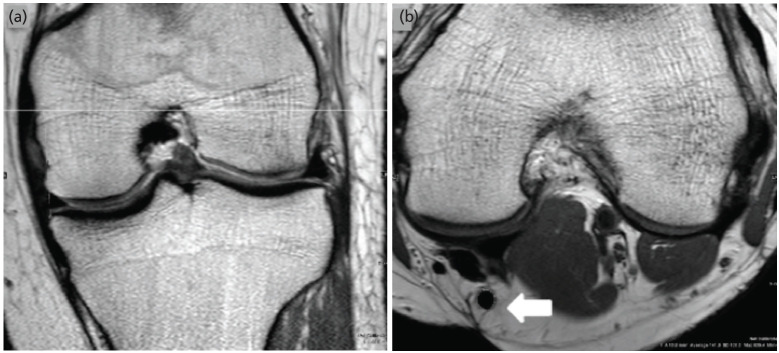
MRI coronal and sagittal views showing the method of estimation of the cross-sectional area of Semitendinosus tendon. The corresponding (a) PD coronal and (b) PD axial section showing the cross-section of Semitendinosus (white solid arrow) at the maximum tendon diameter was recorded using the ROI tool.

The cross-section area (CSA) of the semitendinosus tendon was calculated on the axial images after determining the section using cross hair tool corresponding to the level 3cm from the joint line on the coronal image^9^. The selected image was then magnified once (x2 view) to improve visualisation and to remove the possibility of measuring tissue with signal consistent with either muscle or other soft tissues. The CSA was then calculated by using region of interest (ROI) tool available in the PACS software [Centricity Tm V7.0; GE Healthcare] by placing on the widest dimension of the semitendinosus tendon in the preselected image. This tool automatically determines the CSA (mm^2^). Two readers independently measured each CSA. The first reader was the senior musculoskeletal radiologist with an experience of more than 10 years and the second reader was an Orthopaedician with an experience of 6 years (Fig. 3).

For continuous data, the descriptive statistics Mean, SD was reported. Number of patients and percentage was presented for categorical data. The Pearson correlation coefficient was used to find the relationship between continuous variables. The scatter plot was presented visually to understand relationship between continuous variables. Intra-class Correlation Coefficient was used to assess the reliability of the measurement between the radiologist and surgeon. Bland Altman plot was used to assess systemic difference and scatter of the values. It was plotted as the difference between paired measurements against their mean value. The diagnostic test measures sensitivity, specificity, positive and negative predictive value, positive and negative likelihood ratio were estimated. The area under the curve (AUC) was calculated the Receiver Operating characteristic Curve (ROC) analysis. All tests were two-sided at α=0.05 level of significance. All analyses were done using Statistical Package for Social Sciences (SPSS) software Version 21.0 [Armonk, NY: IBM Corp].

## Results

A total of 69 patients were included in the study, having met the criteria of which 64 were males and 5 females with ages ranging from 17–47 years. A sporting associated injury was the most common cause for an ACL injury in 31 of the 69 patients. Twenty-one patients sustained an ACL tear following a Road Traffic Accident and the remaining nine through a twisting injury to the knee because of a fall.

Among the patients operated for the ACL reconstruction, three patients had a PLC injury which was addressed, and one patient had a concomitant PCL injury which was reconstructed using the ipsilateral Quadriceps tendon and one patient had an MCL injury which was reconstructed as well.

A total of 49 patients had some form of meniscal repair along with the ACL reconstruction. One patient had an irreparable meniscus and a partial meniscectomy was performed. There was only one failure in our series of patients needing a revision ACL reconstruction with Quadriceps tendon.

The mean height of the patients in the study was 172.44±8.07cm with the minimum being 153cm and maximum 190cm. There was one patient whose height was not recorded. The mean weight of the patients in the study was 74.13±13.27kg (Table I).

**Table I:** Mean of the anthropometric data, diameter and length of graft obtained intra-operatively and CSA of graft measured separately by a radiologist and surgeon.

Variables	Mean (SD)
Age, (yrs)	26.60 (7.13)
Height, (cms)	172.44 (8.07)
Weight, (Kg)	74.13 (13.27)
BMI, (Kg/m^2^)	24.83 (3.71)
Diameter, (mm)	7.66 (0.66)
Length, (cm)	6.16 (0.39)
ST radiologist CSA, (mm^2^)	12.52 (1.94)
ST surgeon CSA, (mm^2^)	12.28 (2.36)

Fifty-five patients had a time gap of more than one month since the time of injury and the MRI taken for the estimation of the CSA. The minimum CSA of ST to predict an eventual graft diameter of 7.5mm was 10.7mm^2^.

**Fig 4:**
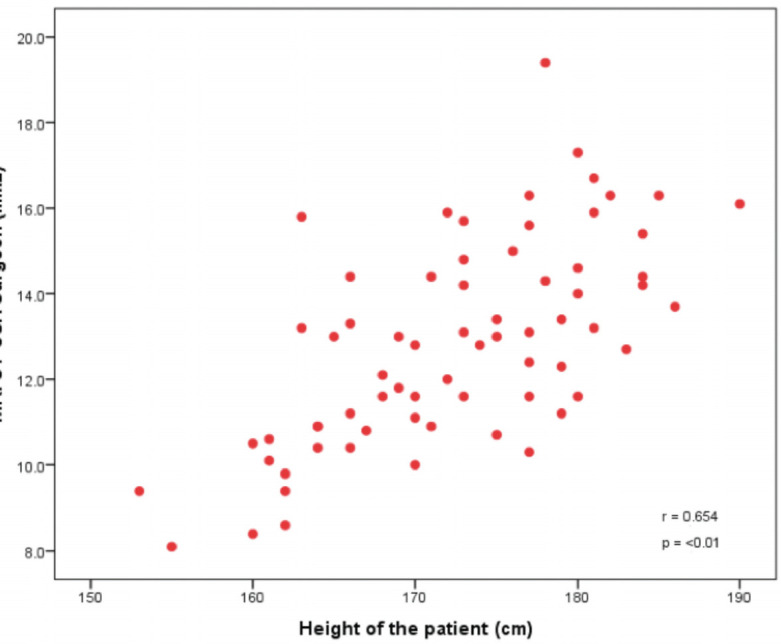
CSA read by the surgeon on the MRI showing a positive correlation with the height of the patient in the above graph.

**Fig 5:**
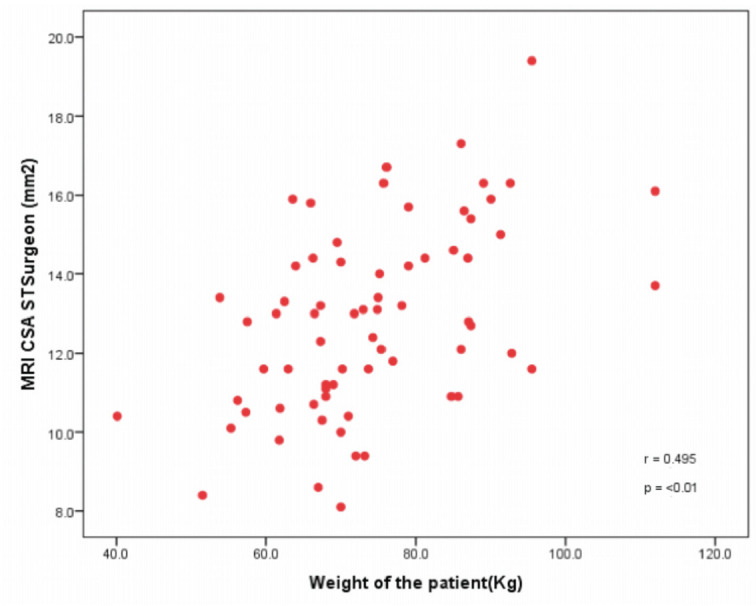
CSA read by the surgeon on the MRI showing a positive correlation with the weight of the patient in the above graph.

There is significant correlation between the graft size obtained intra-operatively and the anthropometric measurements of the patient. The height and weight of the patient positively correlated with the size of the graft as shown in graph below (Fig. 4 and 5).

**Fig 6:**
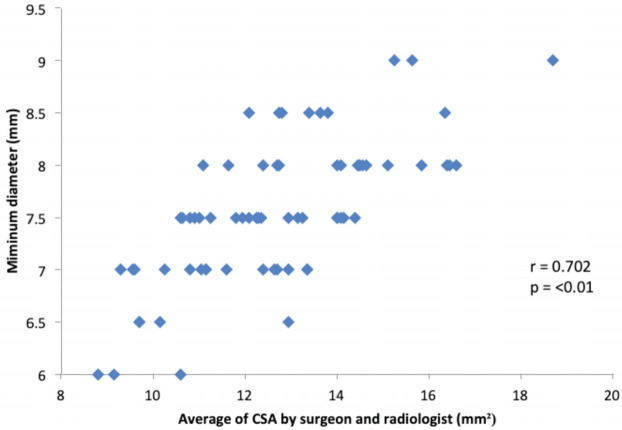
Average of the CSA read by both the radiologist and surgeon and the corresponding intra-operative graft sizes obtained: showing a positive correlation.

The MRI based cross-sectional area measurement showed moderate correlation with the intra-operative graft diameter obtained. (r=0.72, p<0.001) (Fig. 6). For obtaining a graft diameter of at least 7.5mm the cross-sectional area measured on the pre-operative MRI was found to be within the range of 10.7mm^2^ – 15.6mm^2^.

**Fig 7:**
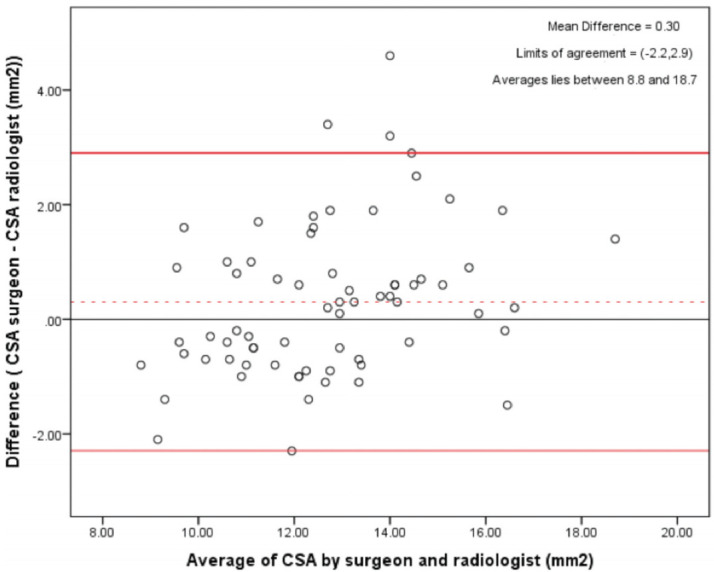
Inter-reader reliability shown to be very high from the graph plotted above.

The Bland and Altman analysis shows the average cross-sectional areas read by the surgeons and the Radiologists shows a mean difference of 0.30 which indicates a high inter-reader reliability (Fig. 7).

The average diameter of the ST graft Cross Sectional Area read by the surgeon was found to be 12.82mm^2^and that read by the Radiologist was 12.52mm^2^. The intra-class correlation coefficient between the radiologist and the surgeon was 0.80, 95% CI (0.70, 0.87) and a p-value <0.001 (Table II).

**Table II:** The intraclass correlation coefficient between the CSA measured by the radiologist and the surgeon with Confidence Interval and P value.

	Intraclass Correlation Coefficient (ICC)	95% Confidence Interval	P-value
MRI ST CSA Surgeon (mm^2^) vs MRI CSA ST Radio (mm^2^)	0.80	(0.70, 0.87)	<0.001

## Discussion

The average cross-sectional area measured by the radiologist and the surgeon were 12.82mm^2^ and 12.52mm^2^ with strong inter-reader reliability. There is a strong correlation between the anthropometric data of the patient and the graft diameter obtained.

The Hamstring graft size is highly variable unlike the BTB or the quadriceps tendon. This variability and uncertainty can lead to suboptimal graft sizes for reconstruction^16^. The ability to accurately predict the eventual graft size by the estimation of the cross-sectional area on the pre-operative MRI will be a useful tool to better prepare the surgeon. The main conclusion that can be made from this study is that the preoperative MRI CSA of the ST can accurately predict the eventual intra-operative graft size obtained. This is in accordance with the previously published literature.

The present analysis results support the previous systematic review findings by Agarwal *et al*, which suggested the pre-operative MRI assessment of both QT and BPTB autografts most highly correlates with intra-operative measurements of autograft diameter and a moderate correlation exists in the predictability of Hamstring Tendon graft^16^. Their conclusions were based on pooled data from four level II studies, six level III studies, and three level IV studies. This comparison further strengthens the applicability of pre-operative MRI as a useful estimator tool.

The minimum CSA on the pre-operative MRI to get a Hamstring graft size of 7mm was 10.7mm^2^ from our study. There was a linear relationship between the CSA and the eventual graft diameter. The strong inter-reader reliability shows that the measurement can be made by the surgeon and obviates the need for a specialist radiologist to measure the CSA of the ST on the pre-operative MRI.

This study also shows a strong positive correlation between anthropometric data, with the height of the patient showing the strongest association. This is consistent with the other studies such as Thwin *et al*^13^. However, this is the first study to be conducted on the South-Asian population.

Ethnic differences in the size of tendons have been shown by a study published by Goyal *et al*^14^. He concludes that the Indian demographic population will often give a diameter lesser than 8mm. In our study population, about 23% of the patients had a graft size of 7mm or less.

Eight patients in our group had a graft diameter at the time of surgery of 7mm or less. In all these patients they were protected with an additional Internal Bracing technique as described earlier.

Three patients who had a diameter of 7.5mm^2^ also underwent internal bracing, this was done as the patients had an increased BMI, and/or the femoral end diameter was 7mm or less. Beyzadeoglu *et al* concluded that with the MRI measurement technique used in their study, patients with a Gracilis cross-sectional area less than 6.4mm^2^ and a semitendinosus cross-sectional area less than 12mm^2^ are in a high-risk group for graft insufficiency and should be well informed about other graft options before surgery^17^.

The reproducibility of the measurement by the surgeon was a strong point in this study. Another strong point in this study is the demographic population constitutes the South-Asian population which has not been investigated in other similar published studies.

For the limitation of the study is the limited sample size in the study. As this is a retrospective study, there is the possibility of a selection bias.

## Conclusion

Pre-operative MRI can be a useful tool to predict the graft diameter. This coupled with the anthropometric data of the patient can be used as an adjunct to estimate the probable graft diameter. Thus, the surgeon can be better prepared for the surgery and can seek alternate graft options if the graft size is deemed inadequate pre-operatively.

## References

[1] Sood M, Kulshrestha V, Sachdeva J, Ghai A, Sud A, Singh S (2020). Poor Functional Outcome in Patients with Voluntary Knee Instability after Anterior Cruciate Ligament Reconstruction.. Clin Orthop Surg..

[2] Shaerf DA, Pastides PS, Sarraf KM, Willis-Owen CA (2014). Anterior cruciate ligament reconstruction best practice: A review of graft choice.. World J Orthop..

[3] Schuette HB, Kraeutler MJ, Houck DA, McCarty EC (2017). Bone-Patellar Tendon-Bone Versus Hamstring Tendon Autografts for Primary Anterior Cruciate Ligament Reconstruction: A Systematic Review of Overlapping Meta-analyses.. Orthop J Sports Med..

[4] Kang H, Dong C, Wang F (2019). Small hamstring autograft is defined by a cut-off diameter of 7 mm and not recommended with allograft augmentation in single-bundle ACL reconstruction.. Knee Surg Sports Traumatol Arthrosc..

[5] Schlumberger M, Schuster P, Schulz M, Immendörfer M, Mayer P, Bartholomä J (2017). Traumatic graft rupture after primary and revision anterior cruciate ligament reconstruction: retrospective analysis of incidence and risk factors in 2915 cases.. Knee Surg Sports Traumatol Arthrosc..

[6] Figueroa F, Figueroa D, Espregueira-Mendes J (2018). Hamstring autograft size importance in anterior cruciate ligament repair surgery.. EFORT Open Rev..

[7] Alomar AZ, Nasser ASB, Kumar A, Kumar M, Das S, Mittal S (2022). Hamstring graft diameter above 7 mm has a lower risk of failure following anterior cruciate ligament reconstruction.. Knee Surg Sports Traumatol Arthrosc..

[8] Hollnagel K, Johnson BM, Whitmer KK, Hanna A, Miller TK (2019). Prediction of Autograft Hamstring Size for Anterior Cruciate Ligament Reconstruction Using MRI.. Clin Orthop Relat Res..

[9] Vardiabasis N, Mosier B, Walters J, Burgess A, Altman G, Akhavan S (2019). Can We Accurately Predict the Quadruple Hamstring Graft Diameter From Preoperative Magnetic Resonance Imaging?. Orthop J Sports Med..

[10] Lubowitz JH (2012). All-inside anterior cruciate ligament graft link: graft preparation technique.. Arthrosc Tech..

[11] Hamada M, Shino K, Mitsuoka T, Abe N, Horibe S (1998). Cross-sectional area measurement of the semitendinosus tendon for anterior cruciate ligament reconstruction.. Arthroscopy..

[12] Ma CB, Keifa E, Dunn W, Fu FH, Harner CD (2010). Can pre-operative measures predict quadruple hamstring graft diameter?. Knee..

[13] Thwin L, Ho SW, Tan TJL, Lim WY, Lee KT (2020). Pre-operative MRI measurements versus anthropometric data: Which is more accurate in predicting 4-stranded hamstring graft size in anterior cruciate ligament reconstruction?. Asia Pac J Sports Med Arthrosc Rehabil Technol..

[14] Goyal T, Paul S, Das L, Choudhury AK (2020). Correlation between anthropometric measurements and activity level on length and diameter of semitendinosus tendon autograft in knee ligament surgery: A prospective observational study.. SICOT J..

[15] Parkes CW, Leland DP, Levy BA, Stuart MJ, Camp CL, Saris DBF (2021). Hamstring Autograft Anterior Cruciate Ligament Reconstruction Using an All-Inside Technique With and Without Independent Suture Tape Reinforcement.. Arthroscopy..

[16] Agarwal S, de Sa D, Peterson DC, Parmar D, Simunovic N, Ogilvie R (2019). Can Preoperative Magnetic Resonance Imaging Predict Intraoperative Autograft Size for Anterior Cruciate Ligament Reconstruction? A Systematic Review.. J Knee Surg..

[17] Beyzadeoglu T, Akgun U, Tasdelen N, Karahan M (2012). Prediction of semitendinosus and gracilis autograft sizes for ACL reconstruction.. Knee Surg Sports Traumatol Arthrosc..

